# Lenalidomide in combination with R-CHOP produces high response rates and progression-free survival in new, untreated diffuse large B-cell lymphoma transformed from follicular lymphoma: results from the Phase 2 MC078E study

**DOI:** 10.1038/s41408-021-00542-z

**Published:** 2021-09-25

**Authors:** Sanjal H. Desai, Betsy LaPlant, William R. Macon, Rebecca L. King, Yucai Wang, David J. Inwards, Ivana Micallef, Patrick B. Johnston, Luis F. Porrata, Stephen M. Ansell, Thomas M. Habermann, Thomas E. Witzig, Grzegorz S. Nowakowski

**Affiliations:** 1 Division of Hematology, Mayo Clinic, Rochester, MN USA; 2 Department of Quantitative Health Sciences, Mayo Clinic, Rochester, MN USA; 3 Department of Laboratory Medicine and Pathology, Mayo Clinic, Rochester, MN USA

**Keywords:** Lymphoid tissues, Cancer therapy

## Abstract

Diffuse large B-cell lymphoma (DLBCL), either concurrent with or transformed from follicular lymphoma (FL) is often excluded from clinical trials. Lenalidomide has response rates of 45% in relapsed transformed DLBCL. Herein we present an analysis of MC078E, a phase II clinical trial testing lenalidomide plus R-CHOP (R2CHOP) for patients with untreated transformed/concurrent DLBCL (NCT00670358). Adult patients with transformed or concurrent DLBCL were included. Patients received six cycles of rituximab, cyclophosphamide, doxorubicin, vincristine, prednisone (R-CHOP) with lenalidomide 25 mg days 1–10 of each cycle. The primary outcome was progression-free survival (PFS) at 24 months. Secondary outcomes were response rates, event-free survival (EFS), and overall survival (OS). Thirty-nine patients were accrued from August 5, 2013 to July 28, 2020 and 33 were eligible by central pathology review. The median age was 64 (24–80) years, 18 (54%) were male, 25 (76%) were concurrent and 8 (24%) were transformed DLBCL. The PFS, EFS, and OS rates at 24 months were 84.4% (CI_95_: 67.2–94.7%), 84.5% (CI_95_: 72.9–98%), and 97.0% (CI_95_: 91.3–100%), respectively. R2CHOP is effective in concurrent and transformed DLBCL. The study supports the inclusion of anthracycline-naive transformed and concurrent DLBCL in future clinical trials of novel immunomodulatory analogues.

## Introduction

Histological transformation of follicular lymphoma (FL) to diffuse large B-cell lymphoma (DLBCL) has a poor prognosis [1, 2]. Outcomes of transformed DLBCL in the pre-rituximab era reported a median overall survival (OS) of 0.6–1 year [3, 4]. Retrospective studies reported that the 5-year OS of transformed DLBCL treated with rituximab, cyclophosphamide, anthracycline, vincristine, and prednisone (R-CHOP) was 40–60% [5–8]. However, patients with indolent lymphoma treated with anthracycline therapy prior to transformation had a worse outcome [5]. These features highlight that transformed DLBCL is a biologically distinct disease.

DLBCL concurrent with indolent FL has similar outcomes to de novo DLBCL but differs in the pattern of relapse with frequent relapse of FL [9]. It is unknown whether concurrent DLBCL represents an early transformation of the indolent lymphoma component or co-development of two independent lymphomas. Molecular genetic studies evaluating for clonal relatedness are needed to make that distinction but are not generally performed as part of the diagnostic work-up of the lymphoma(s). In clinical trials evaluating novel therapeutics, concurrent and transformed DLBCL patients are either in the minority or specifically excluded. Consequently, prospective data on the efficacy of novel regimens in these patients is lacking.

Lenalidomide exerts antiproliferative and immunomodulator actions by ubiquitination and downregulation of lymphoid transcription factors IKZF1 and IKZF3 [10]. Lenalidomide enhances T cell responsiveness to interleukin-2 thereby activating cytotoxic T cells, suppressing regulatory T cells and facilitating differentiation to helper T cells [11]. A pilot study of single-agent lenalidomide for relapsed aggressive NHL enrolled 3 patients with transformed lymphoma and 1 patient responded [12]. This was followed by an international phase 2 study of 33 patients with transformed lymphoma that demonstrated a 45% ORR with 21% CR and a median DOR of 12.8 months [13].

The finding that lenalidomide works synergistically with monoclonal antibody by enhancing antibody-dependent cell-mediated cytotoxicity led to a phase 2 trial of lenalidomide/rituximab that included 9 patients with transformed lymphoma. The ORR was 56% with 33% CR [14–16]. These data provided the rationale to include patients with transformed DLBCL and DLBCL with concurrent FL in MC078E, a phase II clinical trial of lenalidomide plus R-CHOP (R2CHOP) for patients with untreated DLBCL (NCT00670358). An early report of 64 patients with DLBCL showed an ORR to R2CHOP of 98% and a CR rate of 83% [17]. We now report the long-term results of the arm of patients with DLBCL concurrent with or transformed from FL.

## Methods

### Study design and eligibility

This investigator-initiated, single-center, phase 2 study enrolled previously untreated patients with DLBCL. Initially, the trial was designed to include untreated de novo DLBCL. Subsequently, the protocol was amended to include an arm of patients with DLBCL, either concurrent with or transformed from indolent FL. Other key inclusion criteria were: age ≥18 years, determination of DLBCL either concurrent with or transformed from historical FL by central pathology review, Ann Arbor staging II, III, or IV, measurable disease (at least 1 lesion >1.5 cm) by positron emission tomography/computed tomography (PET/CT) or presence of skin nodules of >2 cm size on physical exam, Eastern Cooperative Oncology Group (ECOG) performance status of <2 and adequate organ function. Key exclusion criteria were central nervous system (CNS) involvement, pregnancy, and lactation, the inability of patients of childbearing age to employ adequate contraception, significant cardiac, renal, or liver dysfunction, presence of active malignancy other than DLBCL and FL, history of life-threatening venous thromboembolism, inability to take aspirin, lovenox or warfarin prophylaxis, presence of HIV infection and post-transplant lymphoproliferative disorder.

All patients consented to the study. The study was conducted according to the declaration of Helsinki, approved by the Mayo Clinic institutional review board (IRB) (protocol MC078E), and registered at clinicalTrial.gov (NCT00670358).

### Procedures

Eligible patients received rituximab (375 mg/m^2^), cyclophosphamide (750 mg/m^2^), doxorubicin (50 mg/m^2^), and vincristine (1.4 mg /m^2^) on day 1, prednisone (100 mg) on days 1–5, and lenalidomide 25 mg on days 1–10 of 21-days cycles for up to 6 cycles. Pegfilgrastim was given on day 2 of each cycle to prevent neutropenia. Aspirin 81 mg daily was given to patients not on anticoagulation for VTE prophylaxis. Aspirin was discontinued for platelets <50,000, intolerance, or bleeding complication. Antacids, antiemetic, and tumor lysis prophylaxis were prescribed at the discretion of the treating physician. The use of statins was discouraged while on this study, although not prohibited. A lumbar puncture with cytology of cerebrospinal fluid was performed prior to the beginning of the study regimen at the investigator’s discretion.

The response was assessed with PET/CT after 2 cycles and at the end of treatment according to revised response criteria [18]. First, a follow-up examination was conducted 3 months after completion of study treatment. Subsequent follow-up examinations continued every 3 months for the first year, every 4 months for the second year, and every 6 months for the subsequent 3 years. Follow-up examinations included history and physical exam, evaluation of blood counts, renal function, liver function, and CT chest, abdomen, and pelvis.

Central pathology review was performed by a study hematopathologist (WRM) and included evaluation of hematoxylin and eosin and immunohistochemically stained slides from excisional or needle core biopsies of involved tissues as well as assessment of any accompanying flow cytometric immunophenotyping, fluorescence in situ hybridization, and molecular genetic data on each case. The study diagnosis was based on criteria established by the fourth edition of the World Health Organization classification of tumors of hematopoietic and lymphoid tissues [19, 20].

### Outcomes

The primary endpoint was progression-free survival (PFS) at 24 months, where success was defined as being alive and progression-free at 24 months. If disease status was unknown at 24 months, it was considered a failure. Kaplan–Meier estimates of PFS, defined as the time from registration to either progression or death, were also calculated. Secondary outcomes were overall response rate (ORR), complete response (CR) rate, event-free survival (EFS), and overall survival (OS). OS was defined as the time from registration to death. EFS was defined as the time from registration to progression, death due to any cause, or subsequent anti-lymphoma therapy. Adverse events were recorded according to Common Terminology Criteria for Adverse Events (CTCAE) version 3.0.

### Statistical analysis

A one-stage binomial design was utilized to assess the rate of PFS at 24 months. A total of 35 patients were required to test the null hypothesis that the true rate of PFS at 24 months is at most 50% vs. the alternative hypothesis that it is at least 70%. Success is defined as being alive and progression-free at 24 months. Assuming the number of successes is binomially distributed, this one-stage binomial design has 87% power, with a 9% Type I error rate. At the final analysis, at least 22 successes were required in the first 35 evaluable patients to recommend further testing of this regimen in subsequent studies in this patient population.

Continuous and categorical data were summarized with descriptive statistics. The rate of PFS at 24 months was estimated by the number of patients alive and progression-free at 24 months divided by the total number of evaluable patients and a 95% exact binomial confidence interval was calculated. The Kaplan–Meier method was used to estimate the distributions of time-to-event measures, where differences between groups were assessed using log-rank statistics. Statistical analyses were conducted using SAS version 9.4.

## Results

### Baseline characteristics

**Fig. 1:**
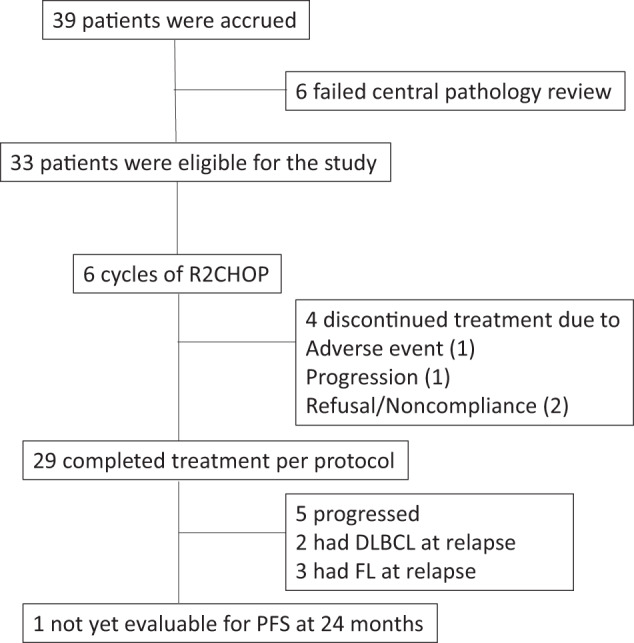
Consort diagram. R2CHOP rituximab, cyclophosphamide, doxorubicin, vincristine, prednisone with lenalidomide, DLBCL diffuse large B-cell lymphoma, FL follicular lymphoma.

Thirty-nine patients were accrued from August 5, 2013 to November 11, 2019 and 33 patients were eligible for study after central pathology review (Fig. 1). Five of the ineligible patients had grade 1–2 FL without DLBCL and 1 had low-grade B-cell lymphoma without DLBCL. Table 1 describes the baseline characteristics of all eligible patients. The median age was 63 (range: 24–80) years. Eighteen (55%) were >60 years old, and 18 (55%) were male. Twenty-five (76%) had concurrent DLBCL and 8 (24%) had transformed DLBCL. Twenty-six (79%) had advanced stage (III-IV). The median number of extranodal sites was 1 (range: 0–4), and 22 (67%) had an extranodal disease. Thirteen (39%) had high-intermediate or high international prognostic index (IPI) (3–5) and 4 (12%) had high IPI (4–5). Twenty-two (67%) had germinal center B-cell (GCB) DLBCL and eleven (33%) had non-GCB DLBCL.

**Table 1:** Baseline characteristics.

Baseline characteristics	Total (*N* = 33)
Age (median, range)	63 (24–80)
Age >60	18 (54.5%)
Sex, *n* (%)	
Male	18 (54.5%)
Performance score, *n* (%)	
0–2	33 (100%)
Diagnosis, *n* (%)	
Composite DLBCL	25 (76%)
Transformed DLBCL	8 (24%)
Stage, *n* (%)	
Advanced stage	26 (79%)
Number of extranodal sites (median, range)	1.0 (0–4)
IPI group, *n* (%)	
0–2	20 (60.6%)
3–5	13 (39.4%)
Age adjusted IPI, *n* (%)	
0–2	32 (97%)
3	1 (3.0%)
COO, *n* (%)	
GCB	22 (67%)
Non-GCB	11 (33%)

*N* number of patients, *%* percentage proportion of patients, *DLBCL* diffuse large B-cell lymphoma, *IPI* international prognostic index, *COO* cell of origin, *GCB* germinal center B-cell type.

Out of 8 patients with transformed DLBCL, 5 had prior systemic therapy that did not include an anthracycline: 3 had immunochemotherapy and 2 had rituximab immunotherapy. Among the 3 that had immunochemotherapy, 2 had bendamustine/rituximab (BR) and 1 had rituximab and cyclophosphamide. Three of the 8 patients had transformed within 1 year of FL diagnosis. Two patients had transformed within 6 months of receiving systemic therapy for FL progression. Three patients had transformed more than 10 years after FL diagnosis.

### Outcomes

#### Efficacy

The median follow-up was 4.4 years (95% CI: 3.7–4.9). Thirty-two (97%) patients received at least 2 cycles of R2CHOP and were evaluable for response. Twenty-nine patients completed all 6 cycles of treatment per study protocol. Reasons for early discontinuations were progression (1), adverse event (AE) (1), refusal of further study treatment (1), and noncompliance (1). ORR in the intent-to-treat population was 97% (32/33, 95% CI: 84–100%), 29 (88%) had CR and 3 had PR.

Thirty-two patients were evaluable for the primary endpoint of PFS at 24 months. One patient had not yet been followed for 24 months at the time of database lock. Twenty-seven patients were alive and progression-free at 24 months (84.4%, 95% CI: 67.2–94.7%). Although the number of evaluable patients was less than what was required per the protocol statistical design, the number of successes per-protocol needed to recommend further study was still met.

**Fig. 2:**
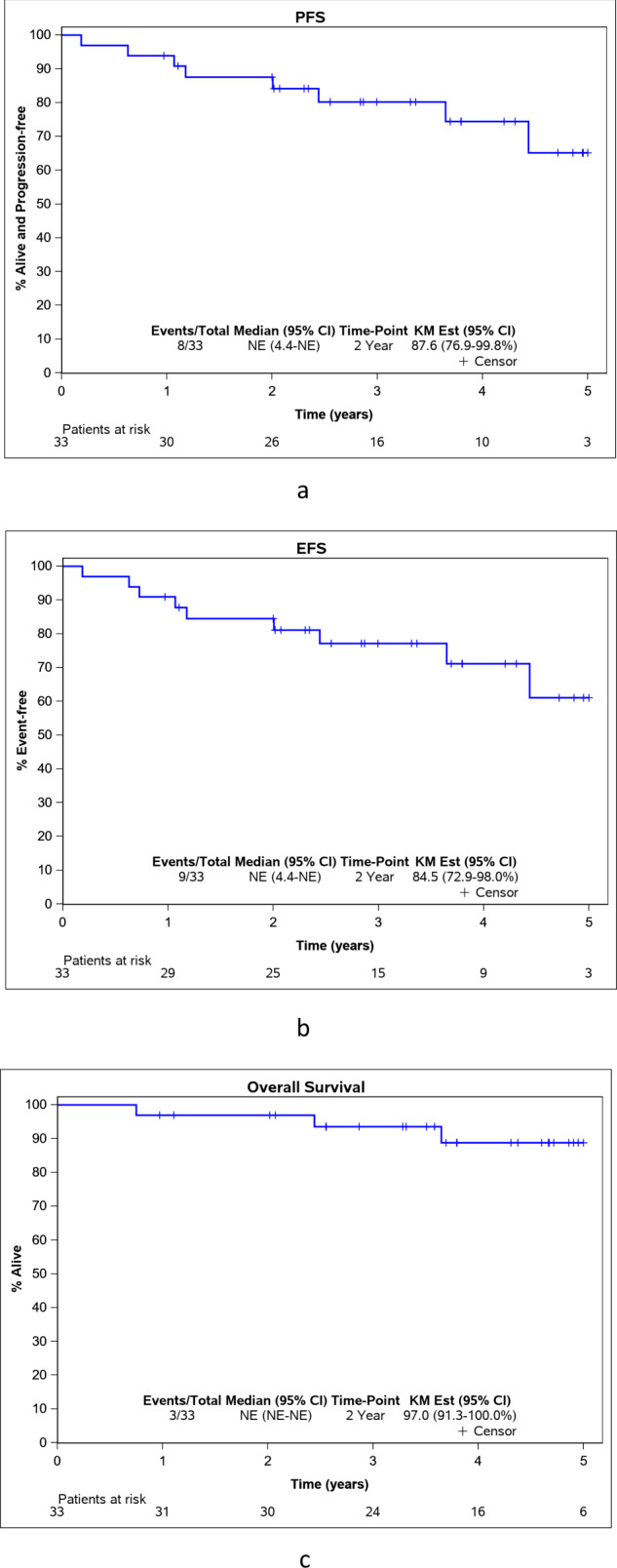
Outcomes for the entire group. **a** Progression-free survival (PFS) for the entire group. **b** Event-free survival (EFS) for the entire group. **c** Overall survival (OS) for the entire group. KM est Kaplan–Meier estimates, NE not estimable, HR hazard ratio.

**Fig. 3:**
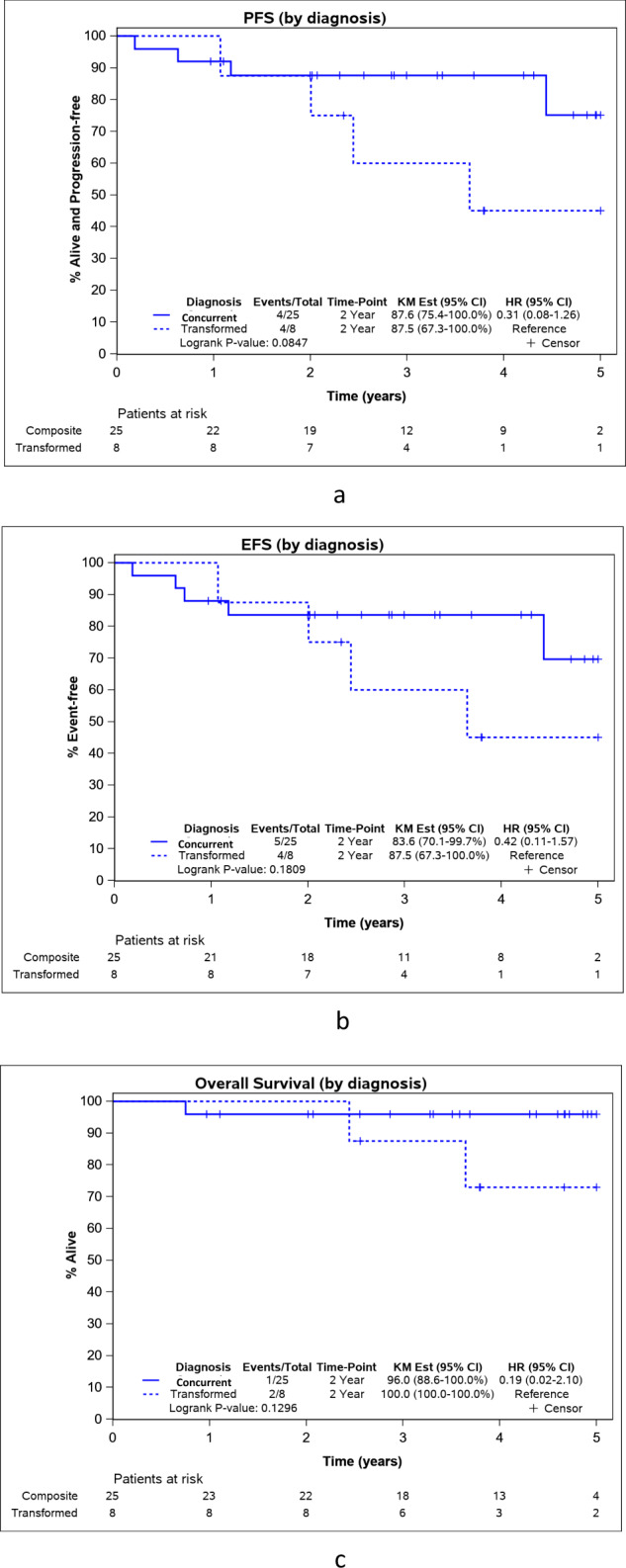
Outcomes by subgroups of concurrent and transformed DLBCL. **a** Progression-free survival (PFS) in the subgroups of concurrent and transformed DLBCL. **b** Event-free survival (EFS) in the subgroups of concurrent and transformed DLBCL. **c** Overall survival (OS) in the subgroups of concurrent and transformed DLBCL. KM est Kaplan–Meier estimates, NE not estimable, HR hazard ratio.

Kaplan–Meier estimates included all 33 patients. PFS at 24 months was 87.6% (95% CI: 76.9–99.8%) (Fig. 2a). Two-year EFS and OS were 84.5% (95% CI: 72.9–98%) and 97.0% (95% CI: 91.3–100%). (Fig. 2b, c). There was no significant difference in PFS of concurrent (PFS24 87.6%, 95% CI 75.4–100%) vs transformed DLBCL (PFS24 87.5%, 95% CI 67.3–100%), with a HR of 0.31 (95% CI 0.08–1.26, *P* = 0.08). EFS and OS were also similar between the two groups (*p*-value: 0.18 and 0.13 respectively) (Fig. 3a–c).

In total, 6 patients had progressed on the study. Three had a relapse of indolent FL (2 at 1 year and 1 at 2 years after registration) and 2 had DLBCL (1 at 2 months and 1 at 1 year after registration) and 1 had a high-grade (triple hit) lymphoma 4 years after registration. Out of 5 patients with transformed DLBCL at study entry who had prior systemic therapy, 2 have relapsed (1 with DLBCL and 1 with FL). Subsequent therapies in patients with relapse of FL included bendamustine/rituximab (BR) (1), rituximab weekly for 4 doses (1), and watchful waiting (1). Subsequent therapies in patients with relapse of DLBCL included: methotrexate with R-DHAP (1), R-ICE (1), and R-GDP (1). Two patients were successfully bridged to autologous stem cell transplant.

#### Safety

All 33 patients received at least 1 cycle of R2CHOP and were included in the analysis of safety. When including adverse events at least possibly related to treatment, 30 (91%) had hematologic AE of grade 3 or above. This included 27 (82%) with neutropenia, 16 (48%) with thrombocytopenia, and 7 (21%) with anemia. Eight (24%) had febrile neutropenia. Table 2 summarizes treatment-related adverse events of all grades. For non-hematologic adverse events, 17 (51.5%) experienced a maximum grade 2, 9 (27.3%) had grade 3 and 3 (9.1%) had grade 4. Eight (24%) had grade 2 and 2 (6%) had grade 3 fatigue. Twenty-four (73%) had alopecia. One patient each had grade 2 fever and grade 2 diarrhea. Three (9%) had grade 2 and 1 (3%) had grade 3 peripheral sensory neuropathy. There were 3 deaths in this study, 1 due to progressive DLBCL, 1 due to acute myeloid leukemia (AML), and 1 due to malignant melanoma. None of the patients died due to adverse event of the study regimen.

**Table 2:** Treatment-related adverse events.

Adverse event^a^	Grade			
	1	2	3	4
	*N* (%)	*N* (%)	*N* (%)	*N* (%)
Hemoglobin decreased	11 (33%)	14 (42%)	7 (21%)	0 (0%)
Leukocyte count decreased	2 (6%)	4 (12%)	8 (24%)	18 (55%)
Lymphocyte count decreased	0 (0%)	5 (15%)	15 (45%)	8 (24%)
Neutrophil count decreased	0 (0%)	5 (15%)	2 (6%)	25 (76%)
Platelet count decreased	11 (33%)	4 (12%)	8 (24%)	8 (24%)
Thrombosis	0 (0%)	0 (0%)	1 (3%)	1 (3%)
Fatigue	0 (0%)	8 (24%)	2 (6%)	0 (0%)
Alopecia	0 (0%)	24 (73%)	0 (0%)	0 (0%)
Rash desquamating	0 (0%)	2 (6%)	0 (0%)	0 (0%)
Constipation	0 (0%)	3 (9%)	0 (0%)	0 (0%)
Dehydration	0 (0%)	1 (3%)	1 (3%)	0 (0%)
Gastritis	0 (0%)	2 (6%)	0 (0%)	0 (0%)
Mucositis oral	0 (0%)	2 (6%)	0 (0%)	0 (0%)
Nausea	0 (0%)	2 (6%)	0 (0%)	1 (3%)
Febrile neutropenia	0 (0%)	0 (0%)	7 (21%)	0 (0%)
Pneumonia	0 (0%)	2 (6%)	1 (3%)	0 (0%)
Sepsis	0 (0%)	1 (3%)	0 (0%)	1 (3%)
Sinusitis	0 (0%)	2 (6%)	0 (0%)	0 (0%)
Skin infection	0 (0%)	1 (3%)	1 (3%)	0 (0%)
Upper respiratory infection	0 (0%)	2 (6%)	0 (0%)	0 (0%)
Blood glucose increased	0 (0%)	2 (6%)	0 (0%)	0 (0%)
Insomnia	0 (0%)	2 (6%)	0 (0%)	0 (0%)
Peripheral sensory neuropathy	0 (0%)	3 (9%)	1 (3%)	0 (0%)
Cough	0 (0%)	2 (6%)	0 (0%)	0 (0%)

*N* number of patients, *%* proportions.

^a^Adverse events with incidence ≥5% and at least possibly related to treatment regimen are presented here.

## Discussion

Our study provides strong evidence of the efficacy of R2CHOP in concurrent and transformed DLBCL in a prospective clinical trial setting, with high response rates, and durable progression-free and overall survival. A significant proportion of our patients had high-risk features with advanced stage and high IPI. Despite that, the response rates seen with R2CHOP speak to the efficacy of lenalidomide in transformed and concurrent DLBCL. Limitations of our study include small sample size and a lack of randomized comparison with R-CHOP. Furthermore, a statistically insignificant trend for lower PFS in 8 patients with transformed DLBCL suggests that the R2CHOP regimen may not lead to sustained disease control in this group, although larger studies are needed to confirm these findings. Nonetheless, the results of our study generate an interesting hypothesis regarding the efficacy of R2CHOP in transformed and concurrent DLBCL and make a strong case for the inclusion of these patients in trials evaluating lenalidomide and other immunomodulators.

Five-year OS of up to 60% after R-CHOP have been reported in retrospective studies of concurrent and transformed DLBCL [5, 6, 9]. Variation in the study population and prior treatment of indolent lymphoma can be responsible for variable outcomes in retrospective studies. Results of our standardized prospective clinical trial compare favorably to outcomes of concurrent and transformed DLBCL treated with R-CHOP in retrospective studies [6, 7, 9, 21].

Lenalidomide combinations have shown encouraging outcomes in treatment-naive DLBCL. R2CHOP has been studied in treatment-naive DLBCL in two randomized trials [22, 23]. The ROBUST trial was a randomized phase 3 trial comparing R2CHOP to R-CHOP in untreated activated B-cell (ABC) type DLBCL[23]. There was no significant difference between R2CHOP and R-CHOP arm, but there was a trend towards better PFS in R2CHOP arm in patients with high-risk and advanced-stage disease [23]. ECOG1412 was a randomized phase 2 trial comparing R2CHOP with R-CHOP in treatment-naive DLBCL [22]. R2CHOP resulted in a 33% reduction in the risk of progression and death compared to R-CHOP [22]. Amongst other postulated reasons for differences in the results of ECOG1412 and ROBUST, one was lenalidomide dosage [24]. ECOG1412 used lenalidomide 25 mg daily days 1–10 of the 21 day of cycle (250 mg total/cycle), higher than the 210 mg/cycle (15 mg/d days 1–14) dosage used in ROBUST [22, 23]. The dose and frequency of lenalidomide used MC078E match that of ECOG1412[22]. Notably, concurrent or transformed DLBCL were excluded from both E1412 and ROBUST. Hence, the efficacy of these novel therapeutic agents in concurrent/transformed DLBCL is uncertain. High and durable response rates presented in our study provide a strong rationale to include concurrent and transformed DLBCL patients in clinical trials evaluating the efficacy of immunomodulators.

The activity of lenalidomide has traditionally been recognized in activated B-cell (ABC) type DLBCL [25, 26]. Notably, studies of the cell of origin of DLBCL included only de novo DLBCL, not transformed/composite DLBCL [27]. Lenalidomide in combination with rituximab has high response rates in both treatment-naive (ORR up to 53%, 2-year PFS 77%) and relapsed refractory FL (best ORR of 78%, 2-year PFS 58%) [28, 29]. Single-agent lenalidomide has shown activity in transformed DLBCL with ORR of 45% and median duration of response of 12.8 months [13]. Thus, there is enough evidence suggesting activity of lenalidomide against germinal center B-cell lymphoma, and inclusion of GCB DLBCL either transformed from or concurrent with indolent FL in clinical trials evaluating regimens of immunomodulators is appropriate.

Patients with FL who receive prior immunochemotherapy and subsequently transform constitute a challenging patient population [5, 30]. Prior anthracycline chemotherapy for indolent FL is associated with poor post-transformation survival of 21% [5]. Patients who receive prior non-anthracycline chemotherapy for indolent FL and subsequently transform have inferior 5-year OS compared to chemotherapy-naive patients in retrospective studies (53% vs 93%, *p* < 0.01) [31]. FL that transformed within 2 years of prior BR had a 2-year OS of 40% [32]. Transformation within 18 months after FL diagnosis predicted poor survival of 22% independent of prior treatment [21]. These results suggest poor prognosis and need to improve frontline treatment in these patients. The optimal treatment of these patients with intensive therapy and consolidative autologous stem cell transplant (ASCT) has not been determined but has been recommended [33]. Prior rituximab exposure for indolent lymphoma decreases the risk of transformation but is also associated with inferior survival post-transformation [21, 30]. In our study, 1 out of 5 patients with prior systemic therapy had a relapse of DLBCL. Out of 3 transformed DLBCL with prior immunochemotherapy, we did not observe DLBCL relapse, and 1 relapse of indolent FL was managed with watchful waiting without any further treatment. Notwithstanding, very small number of patients, results suggest that R2CHOP can potentially provide durable remissions in patients of transformed DLBCL treated with prior non-anthracycline-based chemoimmunotherapy.

Regimens other than R-CHOP have been retrospectively studied in transformed or concurrent DLBCL [34]. Outcomes of transformed and concurrent DLBCL treated with dose-escalated etoposide, cyclophosphamide, doxorubicin, and vincristine with prednisone and rituximab (DA-EPOCH-R) have been reported in retrospective studies [34]. With 2-year PFS of 77% and 81%, respectively, concurrent and transformed DLBCL treated with DA-EPOCH-R had similar outcomes to their de novo counterpart [34]. Outcomes after R2CHOP presented in this study, compare favorably to retrospective data of DA-EPOCH-R. R2CHOP may be a preferable regimen in patients with high-risk transformed or concurrent DLBCL where intensification of R-CHOP is desired and may be potentially more tolerable. Furthermore, evidence of outcomes of concurrent/transformed DLBCL treated with R-CHOP or novel regimen comes from retrospective or observational studies of real-world data. Our study is the only prospective clinical trial, to our knowledge, evaluating the efficacy of a novel regimen in concurrent/transformed DLBCL.

Several novel therapeutic agents are emerging for the treatment of relapsed DLBCL. The addition of anti-CD19 monoclonal antibody tafasitamab to lenalidomide has ORR of 60% and CR of 42.5% and median PFS of 12 months, significantly higher than real-world outcomes of lenalidomide monotherapy [35, 36]. Bispecific antibodies and cereblon modulators are under investigation in relapsed DLBCL with encouraging outcomes [37–39]. Results of our study provide a rationale to include concurrent and transformed DLBCL in future clinical trials of novel therapeutics and provide a benchmark for evaluation of the efficacy of these novel agents for this biologically distinct entity.

## Conclusion

R2CHOP leads to high and durable response rates in anthracycline-naive concurrent and transformed DLBCL in this prospective clinical trial. Concurrent and transformed DLBCL patients should be included in clinical trials evaluating novel regimens; specially lenalidomide and other immunomodulators.
